# Depletion of Na^+^/H^+^ Exchanger Isoform 1 Increases the Host Cell Resistance to *Trypanosoma cruzi* Invasion

**DOI:** 10.3390/pathogens11111294

**Published:** 2022-11-04

**Authors:** João Paulo Ferreira Rodrigues, Leonardo Loch, Thiago Souza Onofre, Nobuko Yoshida

**Affiliations:** Departamento de Microbiologia, Imunologia, e Parasitologia, Escola Paulista de Medicina, Universidade Federal de São Paulo, São Paulo 04039-032, Brazil

**Keywords:** *Trypanosoma cruzi*, Na^+^/H^+^ exchanger isoform 1, host cell invasion, metacyclic trypomastigote

## Abstract

Na^+^/H^+^ exchanger isoform 1 (NHE1), a member of a large family of integral membrane proteins, plays a role in regulating the cortical actin cytoskeleton. *Trypanosoma cruzi*, the agent of Chagas disease, depends on F-actin rearrangement and lysosome mobilization to invade host cells. To determine the involvement of NHE1 in *T. cruzi* metacyclic trypomastigote (MT) internalization, the effect of treatment in cells with NHE1 inhibitor amiloride or of NHE1 depletion was examined in human epithelial cells. MT invasion decreased in amiloride-treated and NHE1-depleted cells. The phosphorylation profile of diverse protein kinases, whose activation is associated with remodeling of actin fibers, was analyzed in amiloride-treated and NHE1-depleted cells. In amiloride-treated cells, the phosphorylation levels of protein kinase C (PKC), focal adhesion kinase (FAK) and Akt were similar to those of untreated cells, whereas those of extracellular signal-regulated protein kinases (ERK1/2) increased. In NHE1-deficient cells, with marked alteration in the actin cytoskeleton architecture and in lysosome distribution, the levels of phospho-PKC and phospho-FAK decreased, whereas those of phospho-Akt and phospho-ERK1/2 increased. These data indicate that NHE1 plays a role in MT invasion, by maintaining the activation status of diverse protein kinases in check and preventing the inappropriate F-actin arrangement that affects lysosome distribution.

## 1. Introduction

The Na^+^/H^+^ exchanger isoform 1 (NHE1) is a member of a large family of integral membrane proteins, which are present in virtually all mammalian tissues and species and regulates intracellular pH, cell volume and transepithelial Na^+^ transport [1,2]. NHE1 is constituted from 12 transmembrane segments with both N and C termini located in the cytosol [3], it is ubiquitously expressed in the plasma membrane and is implicated in cytoskeletal anchoring, contributing to adhesion and migration [4,5]. It was also proposed that the NHE1 functions as an ion exchanger and a regulator of the cortical cytoskeleton, are independent [6]. NHE1 activity is regulated by extracellular signals, mediated by diverse cell-surface receptors [7].

One of the hallmarks of cell invasion by *Trypanosoma cruzi*, the protozoan parasite that causes Chagas disease, is the rearrangement of cortical F-actin and the mobilization of lysosomes to the cell periphery, followed by exocytosis [8,9,10,11]. In a study using a tissue culture-derived trypomastigote (TCT), the *T. cruzi* developmental form, which corresponds to the parasite circulating in the mammalian host bloodstream, was shown to induce the acidification of the target cell cytosol, induce an outward lysosome migration and increased TCT internalization, whereas cytoplasmic alkalinization induced peripheral lysosome depletion and inhibited TCT invasion [8]. *T. cruzi* enters target cells preferentially through their basolateral domains [12], where NHE1 is abundantly expressed in different cell types [13,14,15]. Through its property in regulating intracellular pH and its involvement in actin filament rearrangement, NHE1 may play a role in *T. cruzi* entry into host cells, but that possibility has not been investigated. Of interest is that of all tissues examined for NHE-1 mRNA, the highest levels were detected in the stomach [16]. *T. cruzi* metacyclic trypomastigote (MT), the infective stage from the insect vector, invades the gastric mucosal epithelium, which appears to be the unique portal of entry for systemic *T. cruzi* infection [17]. High efficiency of MT in infecting by the oral route is associated with expression of gp82 [18,19], a stage-specific surface molecule that mediates MT invasion of cultured mammalian cells [20]. Gp82 binds to its receptor, the lysosome membrane-associated protein LAMP2 [21], and induces actin cytoskeleton disruption and lysosome spreading [10,22]. Diverse kinases, such as PKC, ERK1/2 and mammalian target of rapamycin (mTOR), were found to be part of the signaling cascade induced by gp82-mediated MT-target cell interaction. [11,23]. As in cells treated with a drug that triggers extensive F-actin disassembly, PKC and ERK1/2 are highly activated and are associated with altered susceptibility to MT invasion [23], we reasoned that there might be a connection between NHE1 and signaling involving the referred protein kinases. In this study, we investigated the effect of NHE1 inhibitors, as well as NHE1 knockdown on gp82-dependent MT internalization. Additionally, the phosphorylation profile of diverse kinases in NHE1-depleted cells or in cells treated with NHE1 inhibitor was examined. Our results have revealed that inhibition or depletion of NHE1 changes the phosphorylation profile of kinases, such as PKC, ERK1/2, FAK and Akt, and renders the cells more resistant to gp82-mediated *T. cruzi* MT invasion.

## 2. Results

### 2.1. Alkalinization of Host Cell Cytosol Inhibits T. cruzi MT Invasion by Interfering with F-Actin Arrangement and Lysosome Distribution

**Figure 1:**
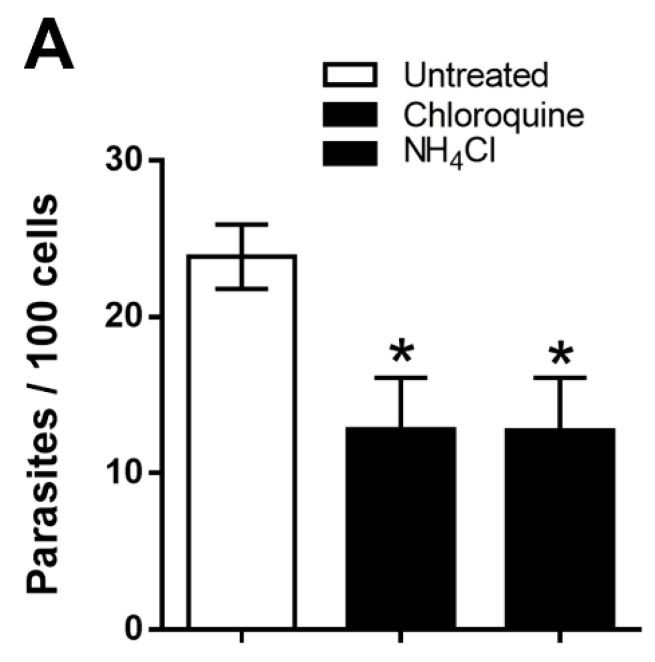

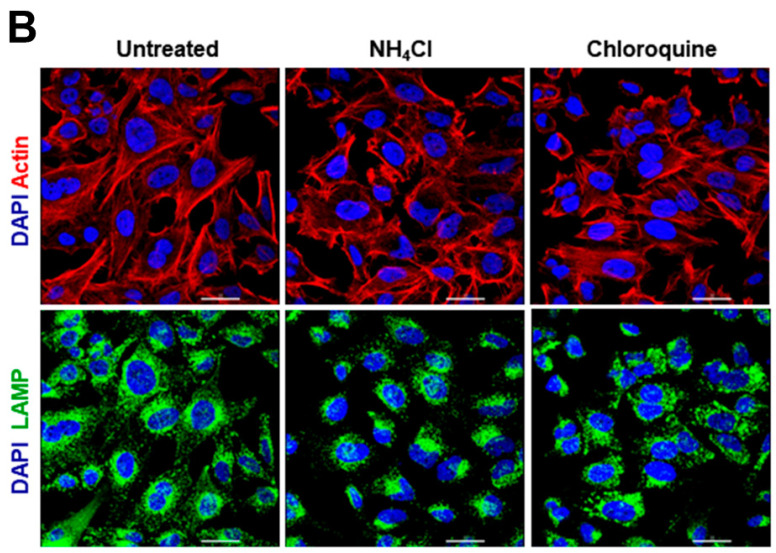
Inhibition of *T. cruzi* MT invasion by treatment of host cells with cytoplasm alkalinization agents that affect F-actin arrangements and lysosome distribution. (**A**) HeLa cells were treated with NH_4_Cl or chloroquine. After removal of drug, the cells were incubated with MT for 1 h, along with untreated cells and the internalized parasites were counted. Data are the means ± SD of four independent assays. Cells pretreated with NH_4_Cl or chloroquine were significantly more resistant to MT invasion (* *p* < 0.005). (**B**) HeLa cells, either untreated or treated with NH_4_Cl or chloroquine, were processed for immunofluorescence to detect actin filaments (red), lysosomes (green) and nucleus (blue). Scale bar = 30 μm.

The effect of host cell cytoplasmic alkalinization on MT invasion was determined. HeLa cells were treated for 30 min with 20 mM NH_4_Cl or 10 µM chloroquine. After the removal of the drug, the cells were exposed to MT for 1 h and the internalized parasites were counted. Cells pretreated by either drug were significantly more resistant to MT invasion (Figure 1A). Using immunofluorescence and a confocal microscope analysis, we checked the profile of F-actin arrangement and localization of lysosomes, which were altered in drug-treated cells, as compared to untreated controls (Figure 1B).

### 2.2. Treatment of Host Cells with NHE1 Inhibitors Reduces the Susceptibility to Invasion by T. cruzi MT

**Figure 2:**
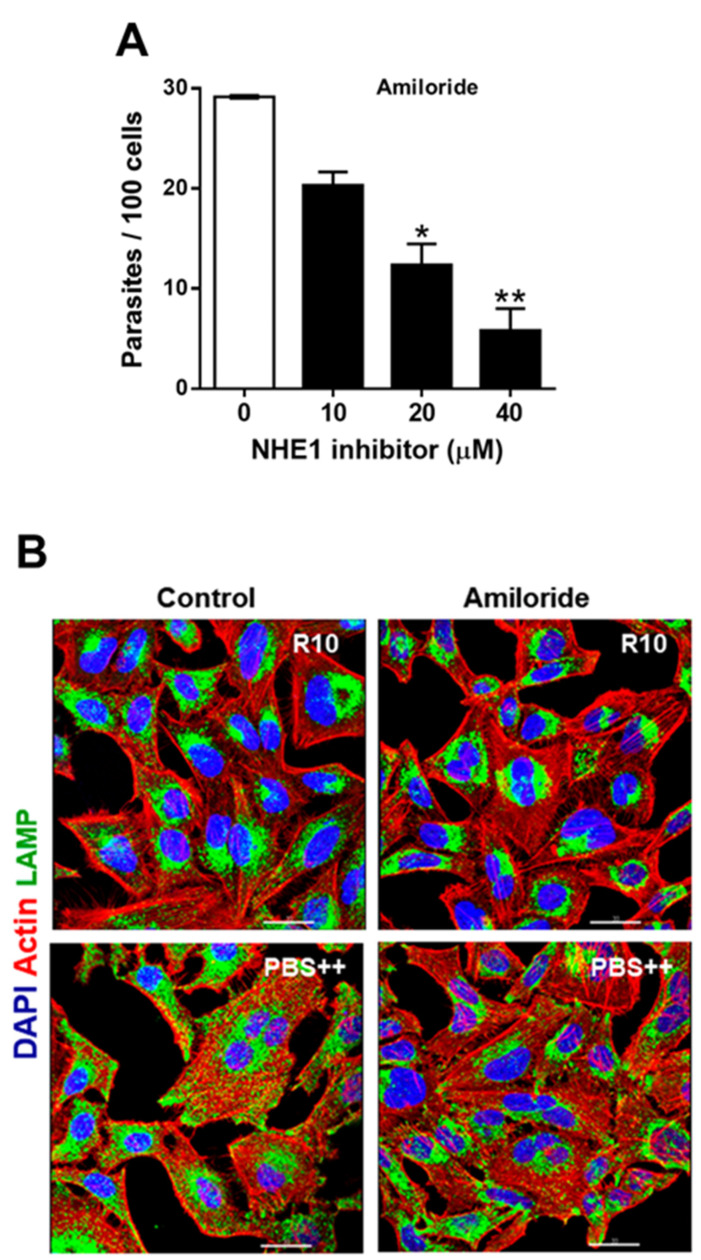
Inhibition of *T. cruzi* MT invasion by treatment of host cells with NHE1 inhibitor amiloride. (**A**) HeLa cells, untreated or pretreated with amiloride at the indicated concentrations, were incubated for 1 h with MT, and then processed for intracellular parasite quantification. Values are the means ± SD of three independent assays. Amiloride significantly reduced MT internalization (* *p* < 0.0005, ** *p* < 0.0001). (**B**) HeLa cells, untreated or pretreated with amiloride at 40 µM were incubated for 30 min in serum-containing RPMI medium (R10) or in PBS^++^. Using immunofluorescence, PBS^++^-induced spreading of lysosomes (green) in untreated cells, and their retention at the perinuclear area in amiloride-treated cells, were observed. Scale bar = 30 µm.

We examined whether amiloride, a commonly used NHE1 inhibitor, affected the susceptibility of host cells to MT invasion. HeLa cells, untreated or pretreated for 2 h with amiloride, at varying concentrations, were incubated with MT for 1 h. Quantification of internalized parasites revealed that MT invasion was significantly inhibited by amiloride in a dose-dependent manner (Figure 2A). Inhibition by amiloride was in the order of 30% at 10 µM and 80% at 40 µM. Pretreatment of HeLa cells with 40 µM amiloride for 1 h was less effective, resulting in about 30% inhibition of MT invasion. To determine whether amiloride affected the host cell lysosome spreading required for MT invasion, HeLa cells, untreated or pretreated with amiloride at 40 µM were incubated for 30 min in RPMI medium containing 10% FBS (R10) or in lysosome mobilization-inducing PBS^++^ medium and processed for immunofluorescence and visualization at the confocal microscope. Upon incubation in PBS^++^, lysosome spreading from the perinuclear area to the cell periphery was observed in untreated cells, but not in cells pretreated with amiloride (Figure 2B). Zoniporide, another selective NHE1 inhibitor, was also tested. At 20 µM and 40 µM, zoniporide showed a significant inhibitory effect on MT invasion, but it was without effect at 10 µM (Supplemental Figure S1A). Pretreatment with zoniporide, at 40 µM, counteracted PBS^++^-triggered lysosome spreading (Supplemental Figure S1B).

### 2.3. Depletion of Host Cell NHE1 Reduces the Susceptibility to MT Invasion by Affecting Actin Cytoskeleton Structure and Lysosome Distribution

**Figure 3:**
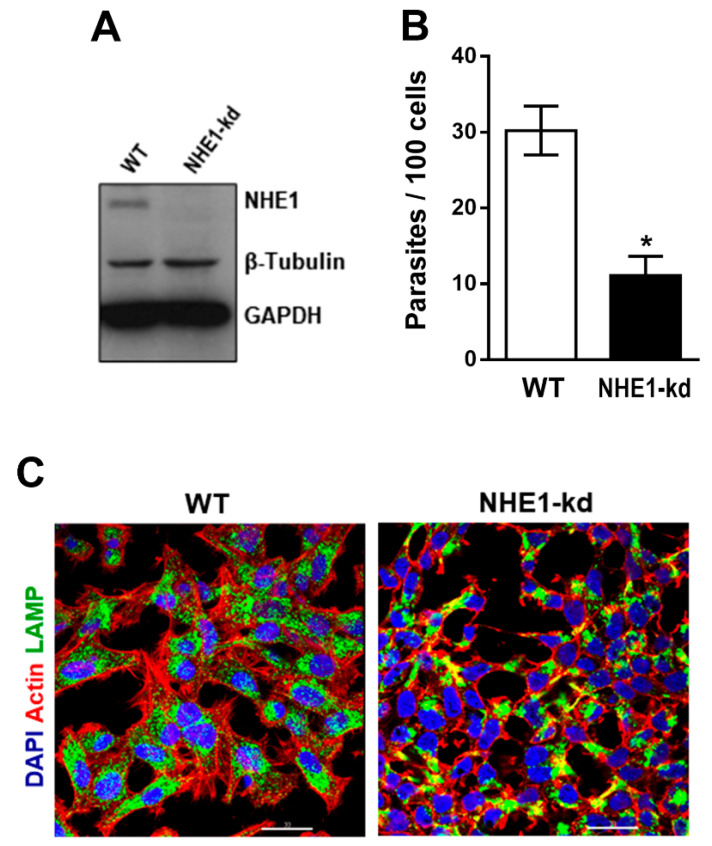
Reduced susceptibility of cells depleted in NHE1 to *T. cruzi* MT invasion. (**A**) HeLa cells were processed for NHE1 knockdown (kd) and analyzed by western blotting. (**B**) Wild type (WT) and NHE1-kd cells were incubated for 1 h with MT, and the internalized parasites were quantified. Values correspond to the means ± SD of four independent assays. MT invasion was significantly diminished in NHE1-kd cells (* *p* < 0.0001). (**C**) WT and NHE1-kd cells were analyzed by immunofluorescence. Representative confocal microscopy image showed altered morphology of NHE1-deficient cells. Scale bar = 30 µm.

**Figure 4:**
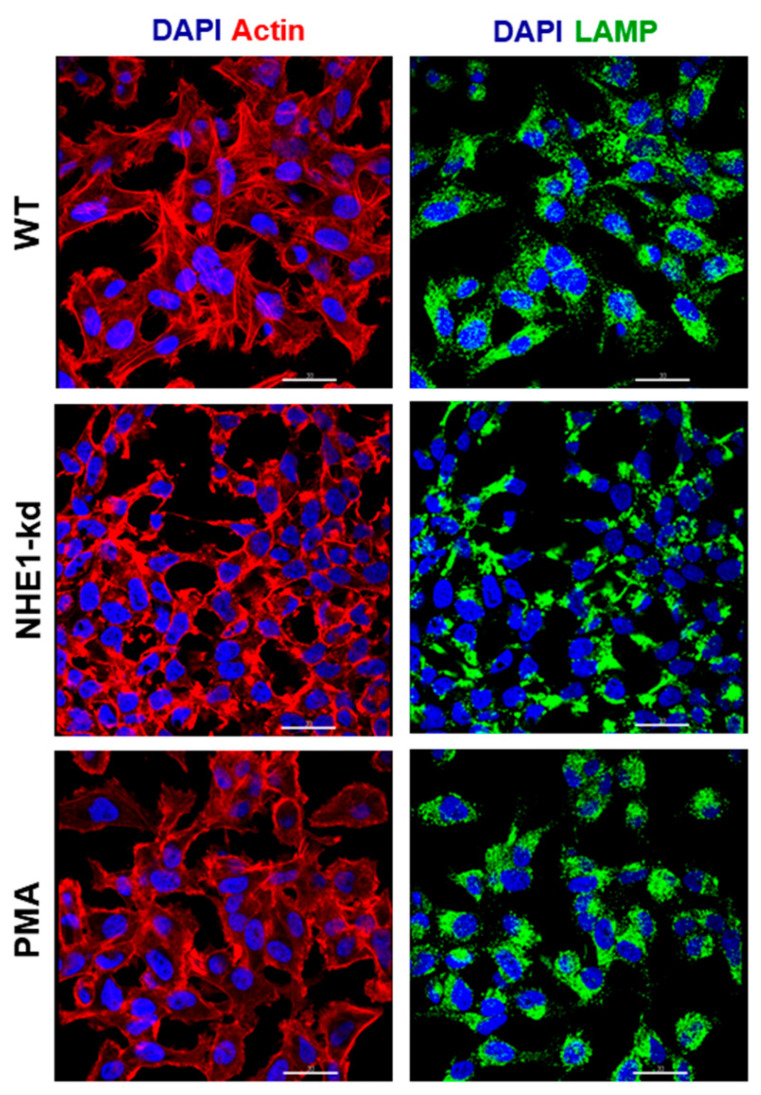
Actin cytoskeleton organization and lysosome distribution in WT, NHE1-depleted or PMA-treated cells. Immunofluorescence assay was performed to analyze the actin cytoskeleton structure and lysosome distribution in WT, NHE1-kd and PMA-treated cells. Representative confocal microscopy images are shown. Scale bar = 30 µm.

The lentiviral transduction methodology was employed to generate HeLa cells depleted in NHE1, using two different target sequences. Upon several attempts, a cell line with reduced NHE1 expression was obtained, as shown by western blot analysis of the detergent-soluble extract (Figure 3A). Cells deficient in NHE1 were examined for their susceptibility to MT invasion. Wild type (WT) HeLa cells and NHE1-depleted cells were incubated for 1 h with MT. Internalized parasite quantification showed significantly increased resistance of NHE1-deficient cells to MT invasion (Figure 3B). The immunofluorescence analysis revealed NHE1-depleted cells were of a smaller size and an altered morphology when compared to WT cells (Figure 3C). We examined the actin cytoskeleton architecture as well as the lysosome distribution in NHE1-depleted cells and in cells treated for 30 min with 100 nM phorbol 12-myristate 13-acetate (PMA), which are also more refractory to MT invasion [11,24]. Differently from control cells, NHE1-depleted cells exhibited thick cortical actin bundles and irregularly distributed compact lysosomes, whereas in PMA-treated cells the loss of stress fibers and a higher accumulation of lysosomes around the nucleus were evident (Figure 4). Lysosomes are of critical importance for gp82-mediated MT internalization. Gp82 binding to host cells induces lysosome spreading and exocytosis [11,22], leading to higher expression of the gp82 receptor LAMP2 on the cell surface [21]. Distribution of lysosomes, such as those observed in NHE1-deficient cells, and the inhibition of lysosome exocytosis in PMA-treated cells [11], presumably impairs the gp82-dependent interaction with host cells.

### 2.4. Phosphorylation Levels of PKC Are Decreased in NHE1-Deficient Cells

**Figure 5:**
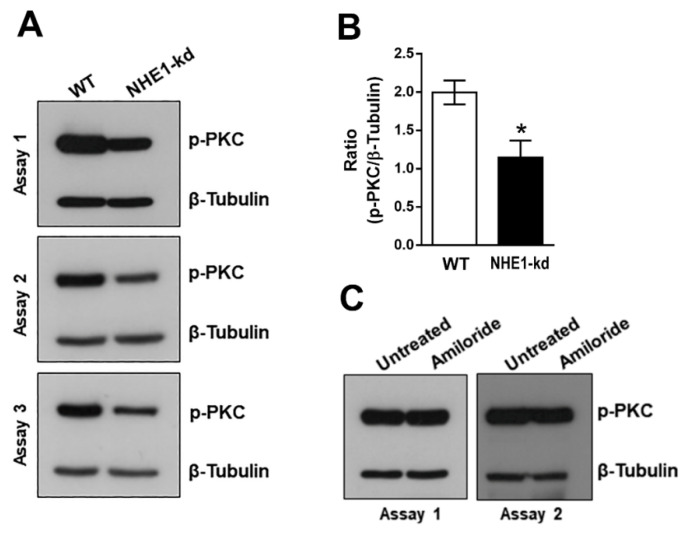
Phosphorylation profile of PKC in NHE1-depleted or amiloride-treated cells. (**A**) Detergent-solubilized extracts from WT and NHE1-depleted cells were analyzed by western blotting to detect phospho-PKC and β-tubulin. (**B**) Western blot bands of phospho-PKC and β-tubulin, shown in (**A**) were quantified and the p-PKC/β-Tubulin ratio was deduced. Values are the means ± of three assays. Decrease in PKC phosphorylation was significant (* *p* < 0.01). (**C**) Extracts from cell treated with amiloride were analyzed as in (**A**).

It was previously shown that PKC is activated in HeLa cells upon interaction with MT or with recombinant gp82 [23]. We examined the phosphorylation profile of PKC in NHE1-depleted and amiloride-treated cells. In repeated assays, the phosphorylation levels of PKC in NHE1-deficient cells were found to be lower, when compared to WT cells (Figure 5A). A significant reduction in phospho-PKC was confirmed by quantifying the western blot bands from three assays, using GelAnalyzer 19.1 software (Figure 5B). In cells treated for 2 h with 40 μM amiloride, the profile of phospho-PKC was similar to that of untreated cells (Figure 5C).

### 2.5. Phosphorylation Levels of ERK1/2 Are Increased in NHE1-Depleted and in Amiloride-Treated Cells

**Figure 6:**
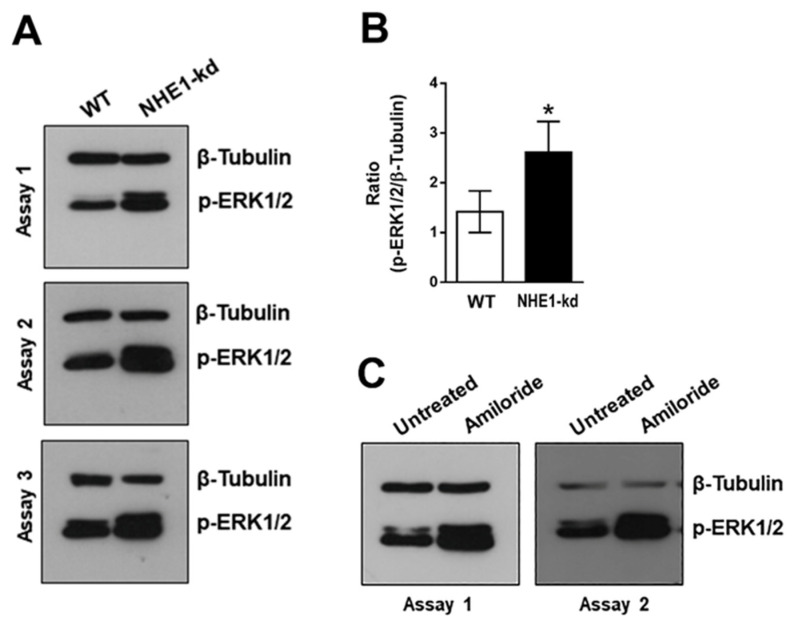
Phosphorylation profile of ERK1/2 in NHE1-depleted or amiloride-treated cells. (**A**) Detergent-solubilized extracts from WT or NH1-depleted cells were analyzed for detection of phosphorylated ERK1/2. (**B**) Western blot bands of p-ERK1/2 and β-Tubulin, shown in (**A**) were quantified and the p-ERK1/2/β-Tubulin ratio was deduced. Values are the means ± of three assays. Increase in ERK1/2 phosphorylation was significant (* *p* < 0.05). (**C**) Extracts from cell treated with amiloride were analyzed as in (**A**).

As activation of ERK1/2 by MT or by recombinant gp82 was also observed [23], we analyzed the phosphorylation profile of ERK1/2 in NHE1-depleted and amiloride-treated cells. In repeated assays, increased ERK1/2 phosphorylation levels in NHE1-deficient cells were detected (Figure 6A) and confirmed by quantifying the western blot bands from three assays (Figure 6B). An increase in ERK1/2 activation was also detected in cells treated with amiloride (Figure 6C).

### 2.6. Akt Is Highly Activated in NHE1-Deficient Cells

**Figure 7:**
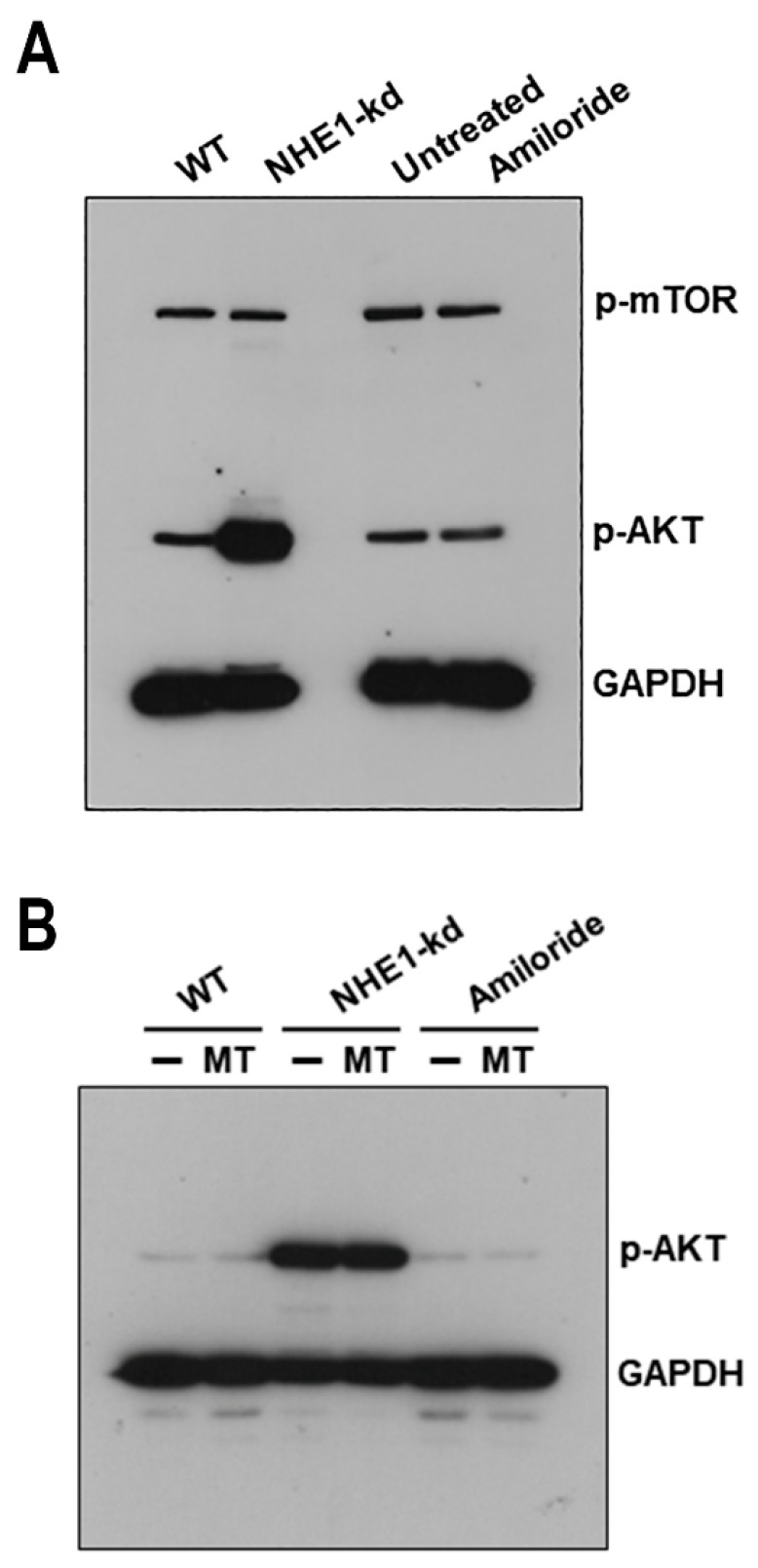
Increased Akt phosphorylation in NHE1-depleted cells. (**A**) Detergent-solubilized extracts from cells, either depleted in NHE1 or treated with amiloride, were analyzed by western blotting for detection of phospho-Akt. Note the increased phosphorylation levels of Akt in NHE1-deficient but not in amiloride-treated cells. (**B**) Detergent-solubilized samples from cells incubated with MT were analyzed. Phosphorylation profile of Akt was not altered by interaction with MT.

We determined the phosphorylation status of serine/threonine kinases Akt and mTOR in NHE1-depleted and amiloride-treated cells. Akt, also called protein kinase B, regulates actin organization [25,26], and mTOR is the mammalian target for rapamycin, a drug that inhibits actin cytoskeleton reorganization [27], which was shown to inhibit lysosome exocytosis and gp82-mediated MT internalization [11,22]. HeLa cells, either depleted in NHE1 or pretreated with amiloride, as well as control cells, were analyzed by western blotting for the detection of phosphorylated Akt or mTOR. Phosphorylation levels of Akt were highly increased in NHE1-deficient cells and were unaltered in amiloride-treated cells; the profile of phospho-mTOR was similar in all cells (Figure 7A). Next, we examined the effect of MT on Akt activation. NHE1-depleted or amiloride-treated cells, as well as control cells, were analyzed by western blotting, after a 30 min incubation with MT. MT did not induce Akt phosphorylation in WT cells or change the profile of phospho-Akt in NHE1-depleted cells or amiloride-treated cells (Figure 7B).

### 2.7. Focal Adhesion Kinase (FAK) Is Dephosphorylated in NHE1-Depleted Cells

**Figure 8:**
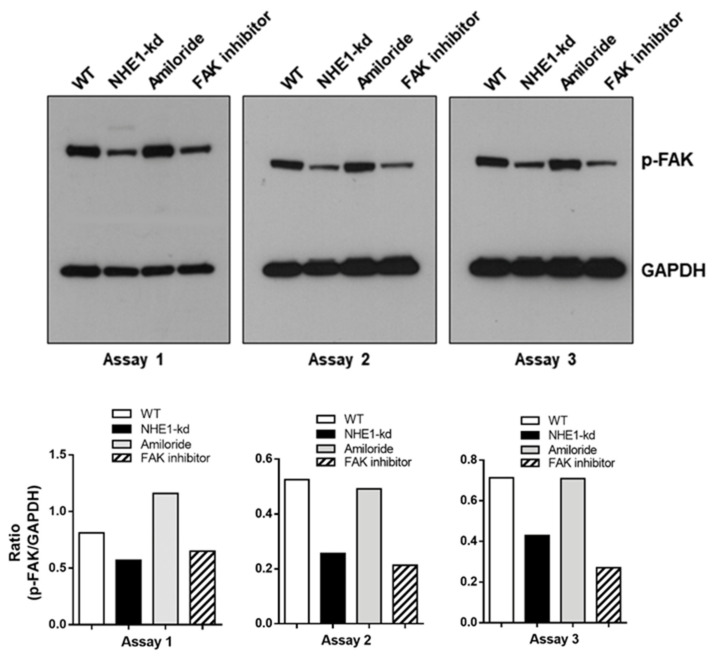
Reduced FAK phosphorylation in NHE1-deficient cells. Detergent-solubilized extracts from cells, either depleted in NHE1 or treated with amiloride or FAK inhibitor, were analyzed for detection of phospho-FAK. Decreased FAK phosphorylation in NHE1-deficient but not in amiloride-treated cells, was confirmed by quantification shown in the lower panel.

FAK, is a cytoplasmic protein tyrosine kinase that regulates F-actin dynamics [28]. Inhibition of FAK was previously shown to reduce the susceptibility of HeLa cells to gp82-mediated MT invasion [29]. Here we examined the profile of FAK phosphorylation in NHE1-depleted and amiloride-treated cells, as well as in cells treated for 45 min with a specific FAK inhibitor PF573228, at 40 μM, in serum-free medium. The western blot analysis of repeated assays revealed a decrease in phosphorylation levels of FAK in NHE1-deficient cells, comparable to that detected in WT cells treated with the FAK inhibitor, whereas amiloride treatment had no effect (Figure 8). Next, we checked whether MT influenced FAK activation. The pattern of FAK phosphorylation was not altered by a 30 min interaction of WT or NHE1-depleted cells with MT (Supplemental Figure S2).

## 3. Discussion

Our study revealed that either inhibition or depletion of NHE1 reduced the host cell susceptibility to gp82-mediated *T. cruzi* MT invasion. We presume that the role played by NHE1 in MT internalization is associated with its function as a regulator of the actin cytoskeleton. Rearrangements of actin filaments influence the distribution of lysosomes, which is a determinant for gp82-dependent MT invasion. Gp82 induces F-actin disruption [10], lysosome spreading and exocytosis [11,22], which increases the availability of gp82 receptor LAMP2 at the plasma membrane [21]. Thus, factors that promote lysosome spreading facilitate gp82-mediated MT invasion, whereas those leading to accumulation of lysosomes in the perinuclear region have the opposite effect [11,22,29]. NHE1-deficient cells differed from WT cells in size, morphology, actin cytoskeleton architecture and lysosome distribution. They were smaller, the cortical actin bundle was thicker, actin stress fibers were not visualized, and clustered lysosomes were irregularly distributed. Such lysosome positioning probably does not facilitate its mobilization and exocytosis. During *T. cruzi* invasion, the lysosomes that are mobilized to the cell periphery are those localized close to the parasite attachment site [30]. Lysosomes clustered around the nucleus, as seen in cells treated with PMA, are also unfavorable for the outward spreading and, accordingly, exocytosis is inhibited in these cells [11].

Gp82-mediated MT interaction with HeLa cells was shown to induce activation of PKC, which translocates to the plasma membrane upon phosphorylation [23]. NHE1-deficient cells exhibited decreased phospho-PKC, when compared to WT cells, which would be compatible with their diminished susceptibility to gp82-mediated MT invasion. However, PKC activation, which induces the disassembly of actin stress fibers, as well as a decrease in peripheral actin network density [31,32], may also render the cells more resistant to MT invasion, as is the case of HeLa cells treated with the PKC activator PMA [11]. In PMA-treated cells, in which highly phosphorylated PKC and a more extensive remodeling of the actin cytoskeleton than that induced by gp82-mediated interaction with MT is seen [23], the lysosome exocytosis is inhibited [11]. It appears from these data that, depending on the degree of PKC activation, different actin rearrangements may ensue, differentially affecting the lysosome distribution. Additionally, to be considered, is the participation of other signaling factors, either in concert with PKC or independent from the PKC pathway. During gp82-dependent MT internalization, an increase in ERK1/2 phosphorylation is induced [23]. Increased phospho-ERK1/2 tightly correlates with spatiotemporal actin dynamics [33] and induces structural and functional changes in NHE1 [34]. We found that phospho-ERK1/2 is increased in NHE1-depleted cells, as well as in amiloride-treated cells, which are more resistant than WT cells to MT invasion. As in the case of PKC, it is not possible to associate the ERK1/2 phosphorylation profile with the pattern of F-actin arrangement or susceptibility to gp82-mediated MT invasion. In HeLa cells treated with the FAK inhibitor for instance, extensive dephosphorylation of ERK1/2 and an altered actin cytoskeleton structure are observed, in association with a higher resistance to gp82-mediated MT invasion [29]. In NHE1-deficient cells, a decrease in FAK activation is observed, comparable to that detected in WT cells treated with the FAK inhibitor. On the other hand, FAK-deficient cells exhibited the same phospho-ERK1/2 profile as WT cells, disassembled F-actin and a higher lysosome spreading and were more susceptible to MT invasion [29]. In contrast to FAK, which was dephosphorylated in NHE1-depleted cells, Akt is highly activated, a finding of note, provided that NHE1 is an Akt substrate required for actin filament reorganization and that NHE1 phosphorylation by Akt increases its H^+^ efflux activity [35].

To date, from the available data, we envisage the following picture. Upon interaction of cells with MT in the gp82-mediated manner, F-actin rearrangements that facilitate lysosome spreading are triggered, in association with the activation of PKC and ERK1/2. By depleting NHE1, the actin cytoskeleton structure is changed in such a way that unevenly distributed lysosomes clusters are formed. These alterations, which render NHE1-deficient cells more refractory to MT invasion, are associated with PKC and FAK dephosphorylation, concomitant with the activation of ERK1/2 and Akt.

## 4. Materials and Methods

### 4.1. Parasites, Mammalian Cells and Invasion Assay

Culture of *T. cruzi* strain CL and enrichment in MT followed the previously described protocol [23]. For MT purification, the parasites were passed in a DEAE-cellulose column, as described [36]. For the invasion assay, human epithelial HeLa cells, obtained from Instituto Adolfo Lutz (São Paulo, SP, Brazil), were incubated for 1 h with MT in R10, at MOI = 10, fixed in Bouin’s solution, stained with Giemsa, and sequentially dehydrated in acetone, acetone:xylol and xylol. Giemsa-stained HeLa cell-coated duplicate coverslips were mounted on glass slides with Entellan (Merck Millipore), and the internalized parasites were counted in a total of 250 cells. This staining procedure allowed to distinguish the effectively internalized parasites from those that are merely adherent, as shown in Supplementary Figure S3. In some assays, we also used PBS^++^ (PBS containing per liter: 140 mg CaCl_2_, 400 mg KCl, 100 mg MgCl_2_.6H_2_O, 100 mg MgSO_4_.7H_2_O, 350 mg NaHCO_3_), as a starvation medium to stimulate lysosome scattering.

### 4.2. Generation of NHE1-Depleted HeLa Cell Lines

For NHE1 knockdown, we relied on RNA interference technology that used lentivirus vectors carrying shRNA directed to *NHE1* [37]). Plasmids containing NHE1 target sequences (Cat No. TRCN0000044648 and TRCN0000044649), from Sigma Aldrich/Merck, were used to produce lentivirus vectors, as previously described [21,29]. HEK293T cells, plated on 100 × 20 mm cell culture dishes (3 × 10^6^ cells per dish), were incubated with a mix of lentiviral packaging and transfection solution [29]. After 48 and 72 h, the cell culture supernatant was harvested and filtered in a 0.45 µm syringe filter. For shRNAi transduction, lentivirus preparation was added to HeLa cells in six well plates (4 × 10^4^ cells/well) in the presence of 4 µg/mL polybrene. After 48 h, the cells were maintained for two weeks, with increasing concentrations of puromycin (0.2–10 µg/mL) for the selection of transduced cells. To check NHE1 depletion, cell extracts were prepared as described [29] and analyzed by western blotting.

### 4.3. Indirect Immunofluorescence Assay

HeLa cells grown on coverslips were fixed and processed as reported previously [29], and then incubated sequentially with mouse anti-human LAMP2 and Alexa Fluor 488-conjugated anti-mouse IgG, diluted in blocking solution for lysosome detection. To visualize the actin cytoskeleton and the nucleus, the cells were incubated with TRITC-phalloidin plus 10 µg/mL DAPI (4′,6-diamidine-2′-phenylindole dihydrochloride) and diluted in blocking solution. After mounting the coverslips in ProLong Gold (Invitrogen), images were acquired using a Leica TCS SP8 laser-scanning microscope (Leica, Germany), at Instituto de Farmacologia e Biologia Molecular (INFAR), Universidade Federal de São Paulo, using a 63X objective and processed and analyzed using Leica LAS AF and Imaris (Bitplane) software.

### 4.4. Antibodies and Reagents

Antibodies for β-tubulin (9F3), GAPDH, phospho-PKCα/β II (Thr638/641), phospho-p44/42 MAPK (ERK1/2) (Thr202/Tyr204), phospho-FAK (Tyr397) and phospho-Akt (Ser473) were from Cell Signaling Technology. Amiloride, zoniporide and the FAK inhibitor PF573228 were from Sigma/Merck. TRITC-phalloidin and Alexa Fluor 488-conjugated anti-mouse IgG were from Thermo Fisher Scientific. Anti-LAMP2 (H4B4) antibody was from Developmental Studies Hybridoma Bank developed under the auspices of the NICHD and maintained by The University of Iowa, Department of Biology, Iowa City, IA 52242.

### 4.5. Statistical Analysis

Student’s *t* test (GraphPad Prism software Version 6.01, GraphPad San Diego, CA, USA) was employed to evaluate significance between groups.

## Data Availability

The data presented in this study are available in this article and Supplementary Material.

## Supplementary Materials

The following supporting information can be downloaded at: https://www.mdpi.com/article/10.3390/pathogens11111294/s1, Figure S1: Inhibition of T. cruzi MT invasion by treatment of host cells with NHE1 inhibitor zoniporide (A) HeLa cells, untreated or pretreated for 2 h with zoniporide at the indicated concentrations, were incubated fo 1 h with MT, and then processed for intracellular parasite quantification. Values are the means + SD of three independent assays. Zoniporide significantly reduced MT internalization (* *p* < 0.05, ** *p* < 0.005). (B) HeLa cells, untreated or pretreated with zoniporide at 40 µM, were incubated for 30 min in serum-containing medium (R10) or in PBS++, and then processed for immunofluorescence for visualization of actin cytoskeleton (red), lysosomes (green), and nucleus (blue). Scale bar = 20 µm. Note the lysosome spreading in untreated cells incubated in PBS++ and the retention of lysosomes at the perinuclear area in zoniporide-treated cells; Figure S2: Reduced FAK phosphorylation in NHE1-depleted cells. (A) Detergent-solubilized extracts from WT or NHE1-kd cells, incubated or not with MT, were analyzed by western blotting for detection of phospho-FAK; Figure S3: Host cell invasion by T. cruzi MT. HeLa cells were incubated for 1 h with parasites and then processed for Giemsa staining. Internalized parasites are indicated by black arrows and those adherent by red arrows. Scale bar = 10 μm. Note the internalized MT surrounded by a clear space. Scale bar = 10 µm.
