# Supplementary material for: Depletion of Na+/H+ Exchanger Isoform 1 Increases the Host Cell Resistance to Trypanosoma cruzi Invasion
Source: Pathogens. 2022 Nov 4;11(11):1294. doi: 10.3390/pathogens11111294 (PMC9698427; doi:10.3390/pathogens11111294)
Supplement: Supplementary file 1 [file pathogens-11-01294-s001.zip › Figure S1.pdf]

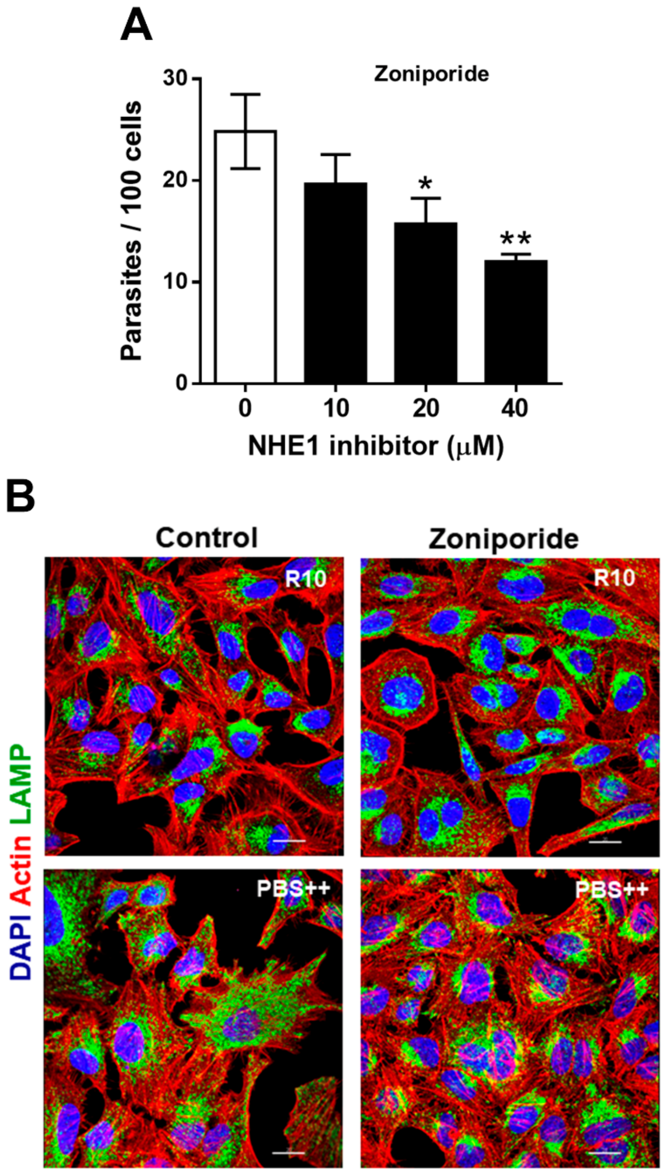

Figure S1. Inhibition of *T. cruzi* MT invasion by treatment of host cells with NHE1 inhibitor zoniporide (**A**) HeLa cells, untreated or pretreated for 2 h with zoniporide at the indicated concentrations, were incubated fo 1 h with MT, and then processed for intracellular parasite quantification. Values are the means ± SD of three independent assays. Zoniporide significantly reduced MT internalization (*P<0.05, **P<0.005). (**B**) HeLa cells, untreated or pretreated with zoniporide at 40 µM, were incubated for 30 min in serum-containing medium (R10) or in PBS^{++}, and then processed for immunofluorescence for visualization of actin cytoskeleton (red), lysosomes (green), and nucleus (blue). Scale bar = 20 µm. Note the lysosome spreading in untreated cells incubated in PBS^{++} and the retention of lysosomes at the perinuclear area in zoniporide-treated cells.
