# Supplementary material for: Depletion of Na+/H+ Exchanger Isoform 1 Increases the Host Cell Resistance to Trypanosoma cruzi Invasion
Source: Pathogens. 2022 Nov 4;11(11):1294. doi: 10.3390/pathogens11111294 (PMC9698427; doi:10.3390/pathogens11111294)
Supplement: Supplementary file 1 [file pathogens-11-01294-s001.zip › Figure S2.pdf]

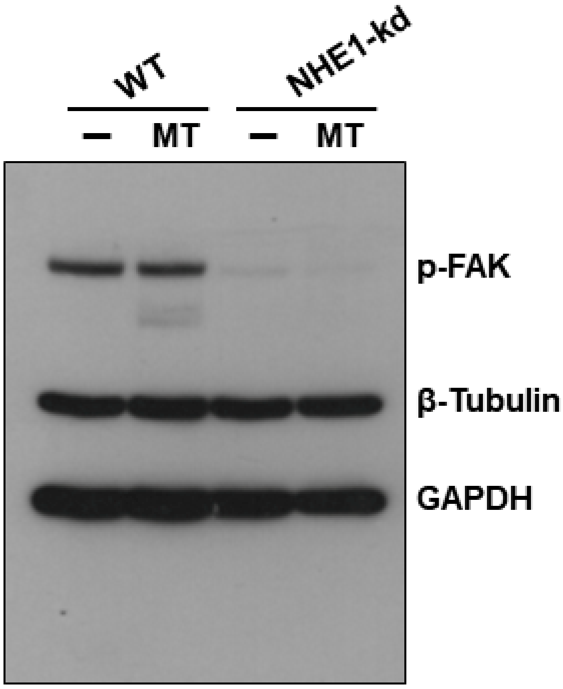

Figure S2. Reduced FAK phosphorylation in NHE1-depleted cells. (**A**) Detergent-solubilized extracts from WT or NHE1-kd cells, incubated or not with MT, were analyzed by western blotting for detection of phospho-FAK.
