# Supplementary material for: Depletion of Na+/H+ Exchanger Isoform 1 Increases the Host Cell Resistance to Trypanosoma cruzi Invasion
Source: Pathogens. 2022 Nov 4;11(11):1294. doi: 10.3390/pathogens11111294 (PMC9698427; doi:10.3390/pathogens11111294)
Supplement: Supplementary file 1 [file pathogens-11-01294-s001.zip › Figure S3.pdf]

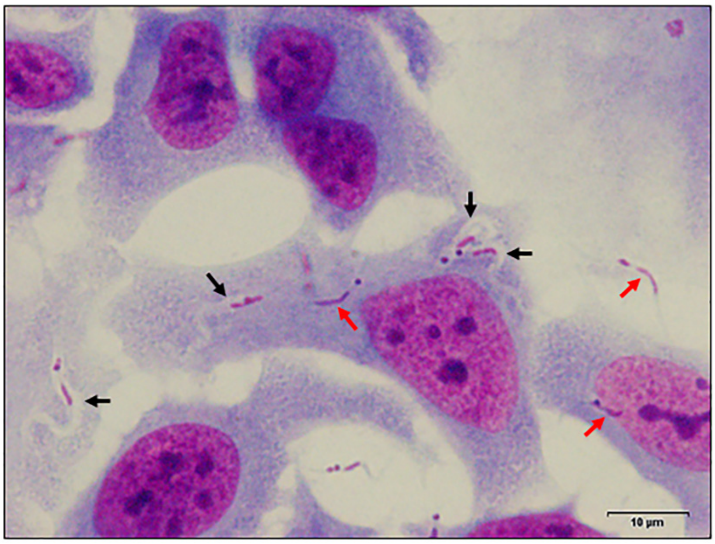

Figure S3. Host cell invasion by *T. cruzi* MT. HeLa cells were incubated for 1 h with parasites and then processed for Giemsa staining. Internalized parasites are indicated by black arrows and those adherent by red arrows. Scale bar = 10 µm. Note the internalized MT surrounded by a clear space. Scale bar = 10 µm.
